# Evaluation of seegene anyplex MTB/NTM real-time detection assay for diagnosis of tuberculous meningitis

**DOI:** 10.1186/s13023-023-03009-5

**Published:** 2024-01-03

**Authors:** Verajit Chotmongkol, Seththawut Kosallavat, Kittisak Sawanyawisuth, Sittichai Khamsai, Narongrit Kasemsap, Nisa Vorasoot, Kannikar Kongbunkiat, Somsak Tiamkao, Prajuab Chaimanee

**Affiliations:** 1 Department of Medicine, Faculty of Medicine, Khon Kaen University, 123 Mittraparp Friendship Road, 40002 Khon Kaen, Thailand; 2 Clinical Laboratory Section, Srinagarind Hospital, Faculty of Medicine, Khon Kaen University, Khon Kaen, Thailand

**Keywords:** Tuberculous Meningitis, Cerebrospinal fluid, Polymerase chain reaction (PCR), Seegeen Anyplex MTB/NTM real-time detection assay

## Abstract

**Background:**

Tuberculous meningitis (TBM) is a common central nervous system infectious disease. Polymerase chain reaction (PCR) assay is a useful method for the rapid diagnosis of TBM. The Seegene Anyplex MTB/NTM real-time detection assay has good sensitivity and specificity for detection of tuberculosis in respiratory specimens, though, data regarding other specimens are lacking. This study aims to define the diagnostic role of Seegene Anyplex MTB/NTM real-time detection assay in TBM in adults.

**Methods:**

This was a retrospective study of 367 adults with symptomatic community acquired meningitis between December 2013 and December 2019. Cerebrospinal fluid (CSF) had been sent for conventional diagnosis, including culture to identify *Mycobacterium tuberculosis*, and Seegene Anyplex MTB/NTM real-time detection assay. Other diagnostic examinations were performed as necessary.

**Results:**

Of the 367 patients in the study, 37 were diagnosed with TBM (14 with definite TBM and 23 with probable TBM). Between the total TBM cases (n = 37) and non-TBM cases (n = 330), clinical sensitivity was 32.4% and specificity was 100%, the positive predictive value was 100%, and the negative predictive value was 93.0%. Between the definite TBM cases (n = 14) and non-TBM cases (n = 330), clinical sensitivity was 50.0% and specificity was 100%, the positive predictive value was 100%, and the negative predictive value was 97.9%.

**Conclusion:**

Due to lack of sensitivity, we suggest Seegeen Anyplex MTB/NTM real-time detection assay should not be used to rule out TBM but is useful for definite diagnosis.

## Introduction

Tuberculous meningitis (TBM) is a common central nervous system (CNS) infectious disease in developing countries. Early diagnosis and treatment with chemotherapy and active management of the complications are of great importance to prevent the irreversible neurologic sequel and death. A definite diagnosis of TBM depends on identifying *Mycobacterium tuberculosis* in the cerebrospinal fluid (CSF) by direct staining or culture. However, the diagnostic yield of CSF smears and cultures is inadequate for diagnosis, owing to the poor sensitivity, and mycological cultures may take up to 6 weeks to yield results [1]. Therefore, TBM diagnosis depends on the clinical manifestations of subacute to chronic meningitis with lymphocytic CSF and low CSF glucose levels. However, other forms of meningitis may mimic TBM. Certain TBM patients may have CSF findings resembling aseptic meningitis. Several rapid tests for TBM diagnosis have been developed. Molecular–based methods, particularly polymerase chain reaction (PCR) assay, are useful methods for the rapid TBM diagnosis. At Srinagarind Hospital, we have recently used Seegene Anyplex MTB/NTM real-time detection assay for the detection of *Mycobacterium tuberculosis* and nontuberculous mycobacteria (NTM). Lim et al. demonstrated that the Seegene Anyplex MTB/NTM real-time detection assay had good sensitivity and specificity for detection of tuberculosis in respiratory specimens [2]. However, data regarding other specimens are lacking. The aim of this study was to investigate the diagnostic role of Seegene Anyplex MTB/NTM real-time detection assay in TBM in adults.

## Materials and methods

### Study population

This was a retrospective study of a consecutive series of 367 adults ((age ≥ 15 years) with symptomatic community acquired meningitis admitted to Srinagarind Hospital, Khon Kaen, between December 2013 and December 2019. Patients were excluded if they had a positive HIV antibody test or had previously received anti-tuberculous drugs before admission. CSF was sent for conventional diagnosis, including white blood cell count, protein and glucose concentrations, Gram stain, Ziehl-Neelsen stain, cryptococcal antigen, and culture to identify *Mycobacterium tuberculosis*. Other diagnostic examinations were performed as necessary. One milliliter of CSF was sent for Seegene Anyplex MTB/NTM real-time detection assay. CSF samples were stored at 4^0^C from the time of collection until analysis, within one week of collection. The laboratory technician was blinded to patients’ diagnoses. The clinicians were unaware of the Seegene Anyplex MTB/NTM real-time detection assay results when the diagnosis was assigned.

### Diagnostic classification

Tuberculous meningitis patients were divided into two groups: definite and probable tuberculous meningitis. Definite TBM was defined if the CSF culture yielded *Mycobacterium tuberculosis* or a positive Ziehl-Neelsen stain. Probable TBM was diagnosed when all of the following were present: sub-acute or chronic meningitis with or without other features of CNS involvement; CSF samples showing pleocytosis with lymphocytic predominance, raised protein concentration, decreased glucose concentration (< 50% of matched plasma glucose), negative bacterial and fungal cultures, and a negative test for bacterial and cryptococcal antigens; and clinical response to anti-tuberculous drugs.

### Statistical analysis

Eligible patients were categorized as TBM or non-TBM. Descriptive statistics were used to present baseline characteristics and clinical factors for both groups. Diagnostic accuracy of the Seegene Anyplex MTB/NTM real-time detection assay for TBM was assessed using sensitivity, specificity, positive predictive value, and negative predictive value calculations. All statistical analyses were performed using STATA software version 10.1 (College Station, Texas, USA).

### Ethical approval

Ethical approval was provided by the Khon Kaen University Faculty of Medicine Ethics Committee as instituted by the Declaration of Helsinki (Approval # HE 611,319).

## Results

Diagnostic results for the 367 patients in the study were as follows; 37 were diagnosed as TBM (14 with definite TBM, confirmed by CSF culture positive for *Mycobacterium tuberculosis*, and 23 with probable TBM), Of the remainder, 49 were diagnosed with cryptococcal meningitis, 29 with aseptic meningitis, 21 with bacterial meningitis, 14 with autoimmune encephalitis, 10 with carcinomatous meningitis, 8 with neurosyphilis, 6 with eosinophilic meningitis, 2 with aspergillus meningitis, and 191 with normal CSF. Clinical presentations and laboratory results are shown in Table 1.

**Table 1:** The clinical manifestations of the patients

Variable	Tuberculous meningitis group (N = 37)	Non-tuberculous meningitis group (N = 330)
Sex, male (%)	59.1	54.5
Mean age	49.5	49.8
**Clinical manifestations**		
Fever (%)	86.4	65.6
Headache (%)	97.7	80.2
Vomiting (%)	43.2	22.6
Neck stiffness (%)	79.5	64.1
Alteration of consciousness (%)	43.2	31.3
Cranial nerve palsy (%)	11.4	18.0
**Laboratory results**		
CSF total WBC count (cell/mm^3^), mean	133.5	276.7
CSF protein concentration (mg/dl), mean	367.8	324.1
CSF sugar concentration (mg/dl), mean	20.1	66.9

CSF = cerebrospinal fluid, WBC = white blood cell

Between the total TBM cases (n = 37) and non-TBM cases (n = 330), clinical sensitivity was 32.4% and specificity was 100%, the positive predictive value was 100%, and the negative predictive value was 93.0% (Table 2). Between the definite TBM (n = 14) and non-TBM cases (n = 330), clinical sensitivity was 50.0% and specificity was 100%, the positive predictive value was 100%, and the negative predictive value was 97.9% (See Table 3).

**Table 2:** The results of CSF PCR between the total tuberculous meningitis cases (n = 37) and non-tuberculous meningitis cases (n = 330)

CSF PCR	Tuberculous meningitis group	Non-tuberculous meningitis group
**Positive**	12	0
**Negative**	25	330
Sensitivity*	32.4% (23.2-32.4%)	
Specificity*	100% (99.0-100%)	
PPV*	100% (71.4-100%)	
NPV*	93.0% (92.0-93.0%)	

CSF = cerebrospinal fluid, PCR = polymerase chain reaction, PPV = positive predictive value, NPV = negative predictive value, *(95% confidence interval)

**Table 3:** The results of CSF PCR between the definite tuberculous meningitis cases (n = 14) and non-tuberculous meningitis cases (n = 330)

CSF PCR	Tuberculous meningitis group	Non-tuberculous meningitis group
**Positive**	7	0
**Negative**	7	330
**Sensitivity**	50.0% (29.7-50.0%)	
**Specificity**	100% (99.1-100%)	
**PPV**	100% (59.3-100%)	
**NPV**	97.9% (97.1-97.9%)	

CSF = cerebrospinal fluid, PCR = polymerase chain reaction, PPV = positive predictive value, NPV = negative predictive value, *(95% confidence interval)

## Discussion

The Seegeen Anyplex MTB/NTM real-time detection assay has been considered a suitable test for diagnostic detection and discrimination between *Mycobacterium tuberculosis* (MTB) and non-tuberculous mycobacteria (NTM). The result’s interpretation is automated and can be reported within 3.5 h of receipt of sample. Our study revealed that between definite TBM and non-TBM cases, Seegene Anyplex MTB/NTM real-time detection assay yielded sensitivity, specificity, positive and negative predictive values of 50.0%, 100%, 100%, and 97.9%, respectively. Between the total TBM cases and non-TBM cases, the study revealed sensitivity, specificity, positive and negative predictive values of 32.4%, 100%, 100%, and 93.0%, respectively. Our results are similar to those of Pai et al. whose systematic review and meta-analysis established a summary accuracy of nucleic acid amplification tests for tuberculous meningitis. The result showed that commercial PCR diagnostic tests for TBM (Roche Molecular Systems, Gen-Probe Inc, and Abbott Laboratories) had a sensitivity of 56% and a specificity of 98%. [3] Xpert® MTB/RIF (Xpert) is a World Health Organization (WHO) recommended, rapid, automated, nucleic acid amplification assay that is also used widely. Kohli, et al’s results to determine the diagnostic accuracy of Xpert revealed a sensitivity of 71.1% and a specificity of 98.0% for TBM. [4]

Lim et al. demonstrated that Seegene Anyplex TB PCR for detection of *Mycobacterium tuberculosis* in respiratory specimens yielded diagnostic sensitivity and specificity values of 87.5% and 98.2% respectively [2]. The lower value of the sensitivity for CSF than the respiratory specimens could be explained by less microorganisms in CSF.

This current study was conducted in routine clinical practice in the endemic area of tuberculosis. The results were similar to another clinical practice study in Korea which has lower incidence of tuberculosis than Thailand [5]. Two PCR tests used in the Korean study had comparable sensitivity rates (38.6% for AdvanSure PCR and 33.3% for COBAS TaqMan PCR) and specificity rates (99.1% for AdvanSure PCR and 98.7% for COBAS TaqMan PCR) as this study for non-respiratory specimens. Note that CSF specimens accounted for 14.2% of non-respiratory specimens (342/2,401). However, these two clinical studies had lower sensitivity rate compared with two systematic reviews: one on extrapulmonary tuberculosis and another one on pathological samples [4, 6]. PCR test for CSF had a sensitivity of 71.1%, while PCR test for pathological samples including TBM had a sensitivity of 70%.

The PCR test for TBM is highly specific and can be performed to diagnose TBM rapidly compared with the CSF culture. Clinicians may use the results of PCR test for TBM as a guide for TBM diagnosis. However, it might be slight differences in sensitivity and specificity among brands of the PCR test. And the sensitivity is generally not quite high.

## Conclusion

Due to lack of detection sensitivity between TBM and non-TBM in our study, we suggest Seegeen Anyplex MTB/NTM real-time detection assay should not be used to rule out the disease but is useful for definitive diagnosis.

## Data Availability

Raw data for Tables 1, 2 and 3 are not publicly available due to individual privacy but are available from the corresponding author on reasonable request.
